# The E3 ubiquitin ligase UBR5 interacts with TTC7A and may be associated with very early onset inflammatory bowel disease

**DOI:** 10.1038/s41598-020-73482-6

**Published:** 2020-10-29

**Authors:** Neel Dhingani, Conghui Guo, Jie Pan, Qi Li, Neil Warner, Sasha Jardine, Gabriella Leung, Daniel Kotlarz, Claudia Gonzaga-Jauregui, Christoph Klein, Scott B. Snapper, Víctor Manuel Navas-López, Aleixo M. Muise

**Affiliations:** 1 Department of Biochemistry, Faculty of Medicine, University of Toronto, Toronto, ON Canada; 2 SickKids Inflammatory Bowel Disease Centre, Research Institute, Hospital for Sick Children, Toronto, ON Canada; 3 Department of Pediatrics, Dr. Von Hauner Children’s Hospital, University Hospital, LMU Munich, Munich, Germany; 4 Regeneron Genetics Center, Regeneron Pharmaceuticals Inc., Tarrytown, NY USA; 5 Division of Gastroenterology, Hepatology and Nutrition, Boston Children’s Hospital, Harvard Medical School, Boston, MA USA; 6 Pediatric Gastroenterology and Nutrition Unit, IBIMA, Hospital Regional Universitario de Málaga, Málaga, Spain; 7 Cell Biology Program, Research Institute, The Hospital for Sick Children, Toronto, ON Canada; 8 Department of Pediatrics, Institute of Medical Science, University of Toronto, Toronto, Canada; 9 Division of Gastroenterology, Brigham and Women’s Hospital, Boston, MA USA

**Keywords:** Gastroenterology, Cell biology

## Abstract

Very early onset inflammatory bowel disease (VEOIBD) denotes children with onset of IBD before six years of age. A number of monogenic disorders are associated with VEOIBD including tetratricopeptide repeat domain 7A (TTC7A) deficiency. TTC7A-deficiency is characterized by apoptotic colitis in milder cases with severe intestinal atresia and immunodeficiency in cases with complete loss of protein. We used whole exome sequencing in a VEOIBD patient presenting with colitis characterized by colonic apoptosis and no identified known VEOIBD variants, to identify compound heterozygous deleterious variants in the Ubiquitin protein ligase E3 component N-recognin 5 (*UBR5*) gene. Functional studies demonstrated that UBR5 co-immunoprecipitates with the TTC7A and the UBR5 variants had reduced interaction between UBR5 and TTC7A. Together this implicates UBR5 in regulating TTC7A signaling in VEOIBD patients with apoptotic colitis.

## Introduction

Very Early Onset Inflammatory Bowel Disease (VEOIBD) may be associated with monogenic disorders^1–11^. Recent studies have demonstrated that approximately 3% of Pediatric IBD patients have a monogenic cause for their disease and younger age at diagnosis is a risk factor^12^.

Previously, we and others identified TTC7A deficiency as a cause of severe intestinal disease^1, 13–18^. Over 50 patients have been identified with pathogenic variants in *TTC7A* associated with a heterogeneous array of phenotypes involving the intestine and immune system^1, 13–25^. VEOIBD patients with *TTC7A* variants have apoptotic enterocolitis and functional studies show loss of interaction with Phosphatidylinositol 4-kinase Type III Alpha (PI4KIIIα) to be the causative factor^1^. PI4KIIIα is involved in the production of phosphatidylinositol 4-phosphate (PI4P) at the plasma membrane (PM)^26,27^ with the help of TTC7A and FAM126A which scaffold PI4KIIIα from endoplasmic reticulum to PM where the complex also interacts with EFR3A/B^28,29^. We also identified Ubiquitin protein ligase E3 component N-recognin 5 (UBR5) as a Tetratricopeptide Repeat Domain 7A (TTC7A) interacting protein using tandem mass spectrometry^1^.

UBR5 is a E3 ubiquitin ligase that has been implicated in several cellular process such as the regulation of DNA damage^30^, metabolism^31^, transcription^32^, and apoptosis^33^. *Ubr5*^*-/-*^ mice fail to grow beyond the E10.5 embryonic development stage and knockout of *hyd* (‘hyperplastic discs’) and UBR5′s homologue in *Drosophila melanogaster*, results in lethality in the pupal or larval stages^34^. Recent studies have described UBR5 as an oncogene in colorectal cancer (CRC)^35–37^. *UBR5* was also found to be more often mutated in a higher percentage of cases resulting in transitioning from IBD to CRC compared to only CRC cases^38^.

Here, we identified bi-allelic damaging variants in UBR5 in a VEOIBD patient who presented with severe colonic disease. Functional studies demonstrate that UBR5 co-immunoprecipitates (co-IP) with TTC7A implicating UBR5 in the TTC7A-PI4KIIIα complex signalling.

## Results

### Patient summary

A boy of Spanish ancestry, born to healthy non-consanguineous parents, presented with diarrhea, rectal bleeding and rectal prolapse at age 2 years and 9 months (Supplementary Figure 1A and Table 1 for blood work analysis). He had no extra intestinal manifestations of disease. Colonoscopy and histopathological examination demonstrated patchy inflammatory cell infiltrates with apoptosis leading to a diagnosis of IBD-Unclassified (Supplementary Figure 1B). He was initially treated with intravenous steroids but eventually required oral tacrolimus due to poor response. His treatment was changed to rectal 5-ASA and azathioprine after response to tacrolimus. At 4 years and 10 months age, his disease flared (Pediatric Ulcerative Colitis Activity Index^39,40^ [PUCAI] score of 75 points) with no response to intravenous steroids and was switched to infliximab. Currently, he is maintained with 10 mg/kg/4 weeks infliximab (IFX) and rectal 5-ASA (due to a proctitis unresponsive to IFX) and oral 5-ASA with his most recent endoscopy showing only mild proctitis (Supplementary Figure 1C).

**Figure 1:**
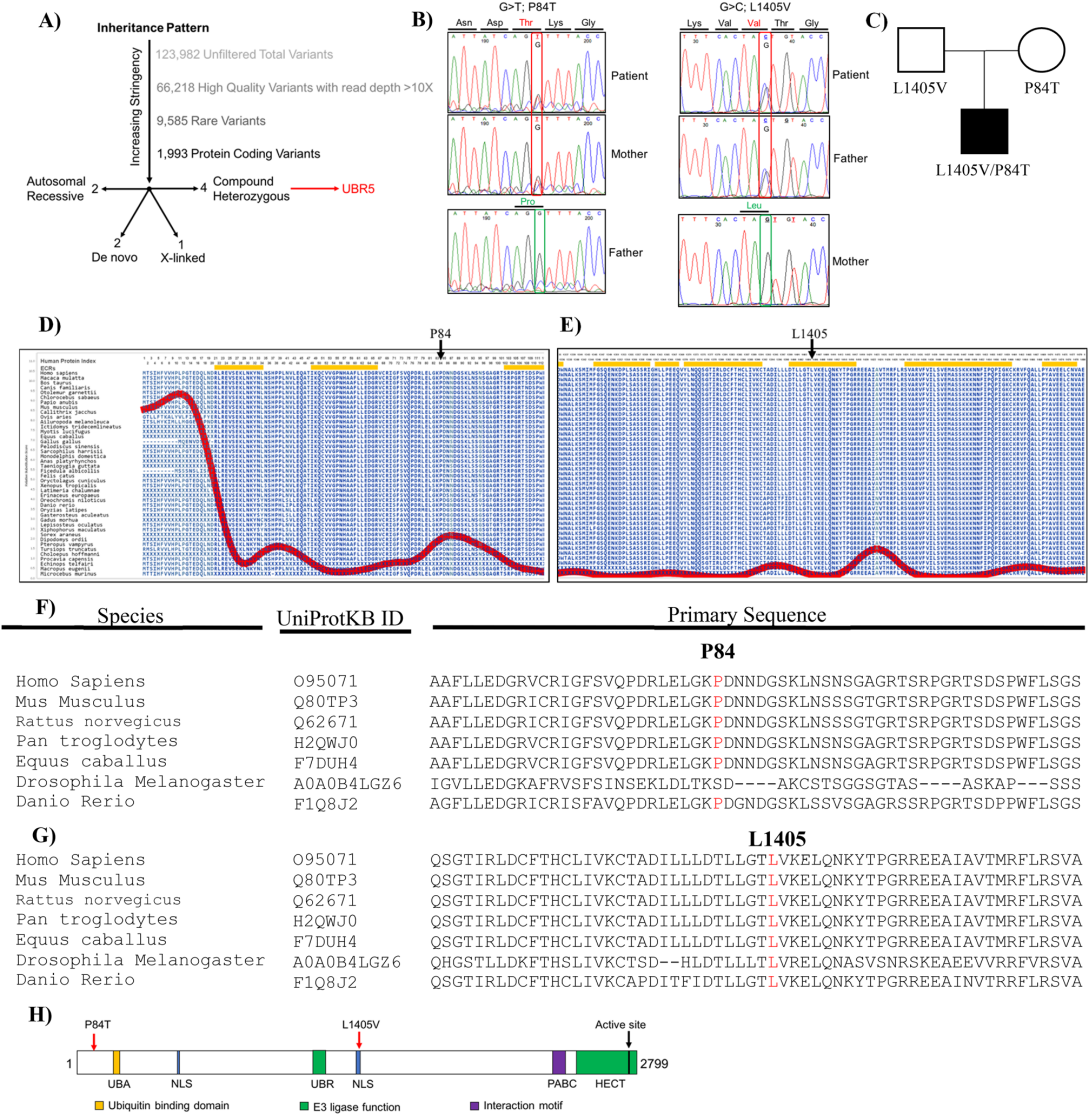
Filtration strategy from Whole Exome Sequencing (WES) for selection of *UBR5* as a disease causing variant and genetic analysis of the trio for UBR5 patient. (**A**) WES of the TRIO identified total of 123,982 variants in the patient. Low-quality variants were removed. Afterwards, common variants with maf < 0.01 from 1000Genomes_phase3^62^ were removed. To isolated potential causative variants, only protein coding variants were included in the inheritance analysis. Finally, variants with CADD > 20 and max maf < 0.01 were identified resulting in various inheritance models such as autosomal recessive, de novo, x-linked, and compound heterozygous. (**B**) Sanger sequencing of the compound heterozygous variants found in the patient and parents. (**C**) Pedigree of the affected patient’s family and the inheritance pattern of the mutations in the patient. Aminode analysis for UBR5 from multiple species shows strong conservation of (**D**) Proline (P) at position 84 and (**E**) Leucine (L) at position 1405. The red line shows amount of sequence substitution at that amino acid position^63^. ECR = evolutionarily constrained region. CLUSTALW multiple species sequence alignment for UBR5 by MUSCLE shows strong conservation of (**F**) Proline at position 84 and (**G**) Leucine at position 1405. (**H**) Location of the mutations on the UBR5 domain architecture. Figure adapted from Shearer et al.^51^. UBA = ubiquitin associated (UBA) domain, UBR = ubiquitin recognin box, NLS = nuclear localization sequences, and PABC = domain homologous to C-terminus of Poly-Adenylation Binding Protein.

### Genetic analysis

Analysis of whole exome sequencing of DNA samples from the families did not identify any known monogenic disorder associated with VEOIBD (see Fig. 1A for analysis strategy and Supplementary Table 2 for full list of potential variants). However, we identified biallelic compound heterozygous nonsynonymous variants in the *UBR5* gene of the patient (Supplementary Table 2 for further information regarding UBR5 mutation including MAF and damaging scores). A variant in exon 4 of *UBR5* [hg38.g.chr8:102360605(G>T); NM_015902:c.250C>A] resulting in a proline to threonine substitution at amino acid position 84 (p.P84T) was inherited from the unaffected mother (Fig. 1B,C). This variant is rare (gnomAD^41^ minor allele frequency (MAF) = 0.000081) and predicted to be deleterious by bioinformatic algorithms, including a PHRED scaled combined annotation dependent depletion^42^ (CADD) score (ver 1.3) of 23.5. A variant *in trans* in exon 33 of *UBR5* [hg38.g.chr8:102294091(G>C); NM_015902:c.4213C>G] was inherited from the unaffected father (Fig. 1B,C) resulting in a leucine to valine substitution at amino acid position 1405 (p.L1405V). This variant has a CADD score of 24.6 and is not present in gnomAD. Both affected residues, P84 and L1405, are highly conserved across species (Fig. 1D–G respectively). While the p.P84T variant is not located in a known domain, the p.L1405V variant is predicted to affect the second nuclear localization sequence of UBR5^43^ (Fig. 1H). A number of VEOIBD databases were searched for other patients with potential UBR5 variants but none were identified.

### Functional studies

#### Immunohistochemistry

**Figure 2:**
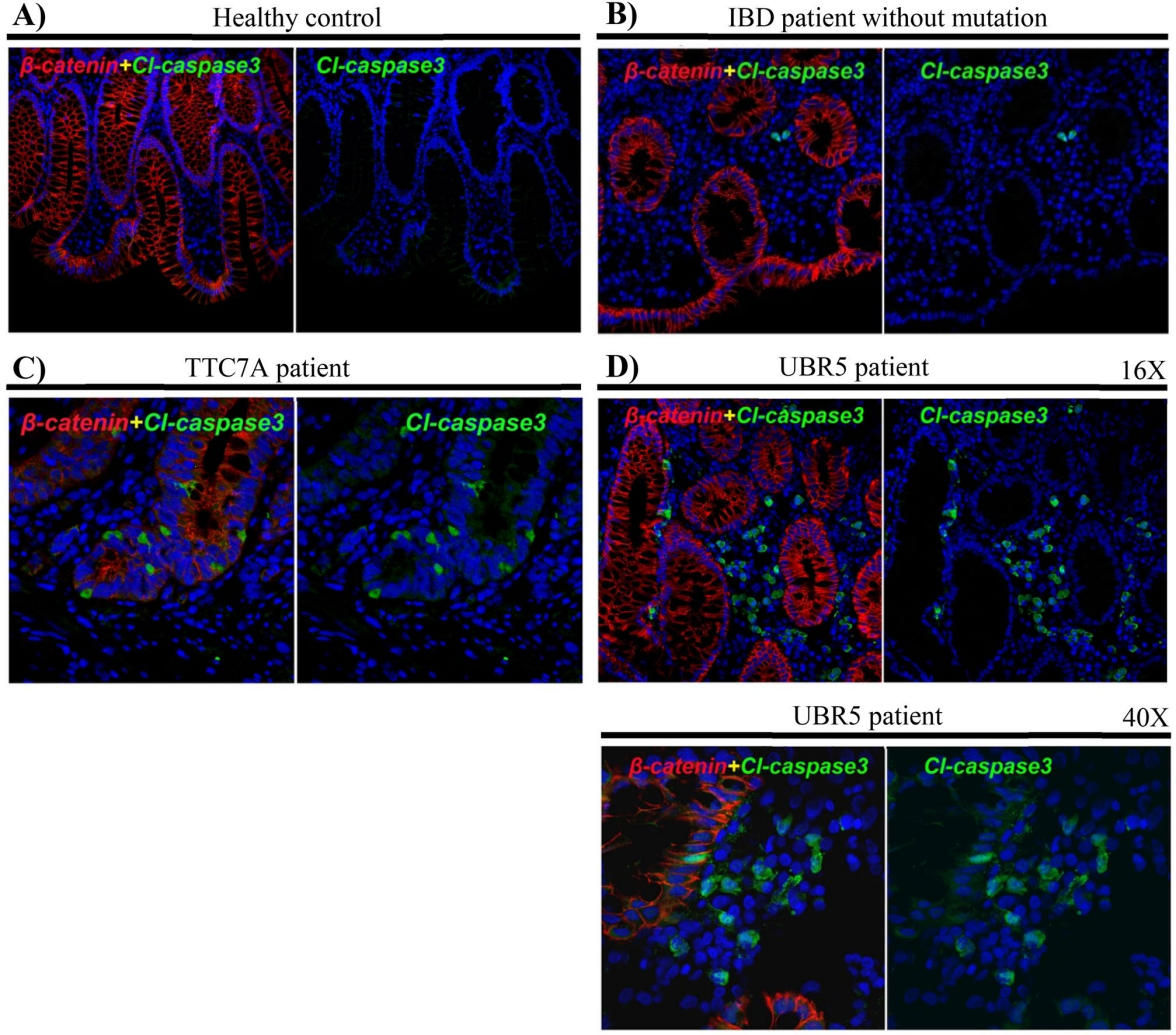
Elevated caspase-3 activity in the UBR5 patient. Immunohistochemistry (IHC) of Formalin-Fixed Paraffin Embedded (FFPE) colon sections from (**A**) healthy control, (**B**) IBD patient without mutations, (**C**) *TTC7A* patient, and (**D**) *UBR5* patient. Cleaved (Cl) caspase-3 is shown in green, β-catenin in red and nuclear counterstaining in blue (RedDot2).

As Hemotoxylin and Eosin staining of the patient’s colonic biopsies (Supplementary Figure 1B) demonstrated increased apoptosis, we further examined apoptosis using immunohistochemistry (IHC) staining for cleaved (Cl) Caspase 3. Dual labeling of colon sections in a healthy control and patient with IBD and with known variants, we observed minimal cleaved(cl)-Caspase 3 positive cells (Fig. 2A,B). In contrast, patients with *UBR5* or *TTC7A* variants had increased cleaved-Caspase 3 positive cells (Fig. 2C,D). In our patient with *UBR5* variants, we observed increased cleaved-Caspase 3 positive cells in the lamina propria (Fig. 2D). In contrast, in the TTC7A patient section (Fig. 2C), the cleaved-Caspase 3 positive cells are only in the epithelium. There is no observed difference in the intensity and architecture for β-catenin between UBR5 patient section (Fig. 2D) and control sections (healthy control + IBD patient sections) (Fig. 2A,B). However, there is a disruption in epithelial layer architecture for β-catenin in TTC7A patient section (Fig. 2C).

**Figure 3:**
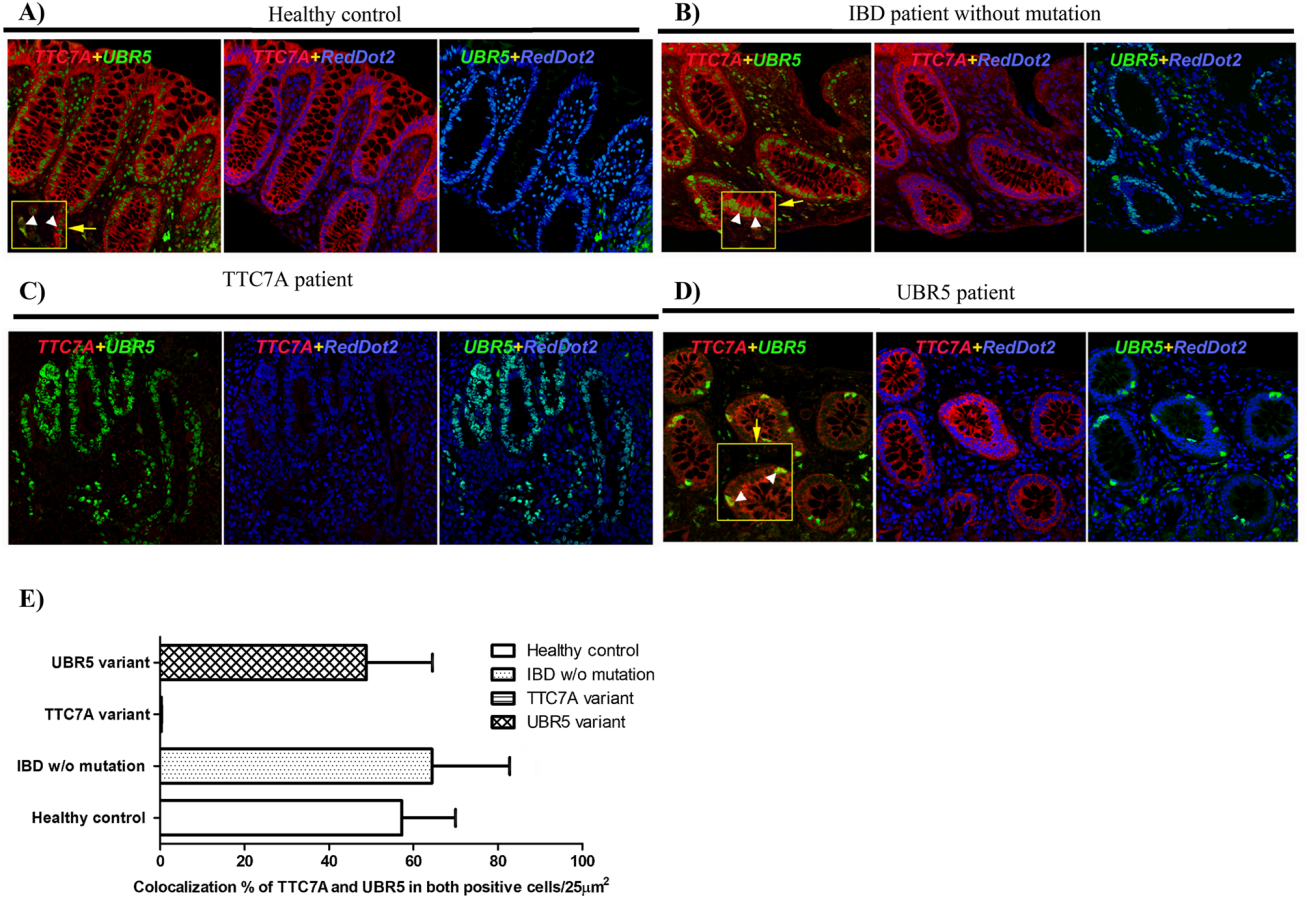
IHC of FFPE colon sections from (**A**) a healthy individual, (**B**) an IBD patient without mutations, (**C**) a patient with *TTC7A* variants and (**D**) a patient with compound heterozygous variants in *UBR5*. UBR5 is shown in green, TTC7A in red and nuclear counterstaining in blue (RedDot2). (**E**) Shows percentage of cells showing colocalization between UBR5 and TTC7A in 100 cells stained positive for both, UBR5 and TTC7A, in selected areas.

IHC staining for UBR5 and TTC7A in a healthy control showed that in the colon, UBR5 localized mainly in the nuclei of immune cells with minimal epithelial expression (Fig. 3A). In colonic sections from an IBD patient without *UBR5* or *TTC7A* variants and a TTC7A-deficiency patient (previously described^44^), we observed upregulation of UBR5 in epithelial cells of the colon (Fig. 3B,C). No colocalization of UBR5 and TTC7A labeling could be observed in the TTC7A patient (Fig. 3E and Supplementary Table 3). Interestingly, colonic sections from our UBR5 variant patient demonstrated a different pattern of localization in the patient, as compared to healthy controls and IBD patients with strong signal intensity for UBR5 aggregated in the epithelial indicative of intra-epithelial lymphocytes (Fig. 3D,E).

#### Identification of TTC7A as a binding partner of UBR5

**Figure 4:**
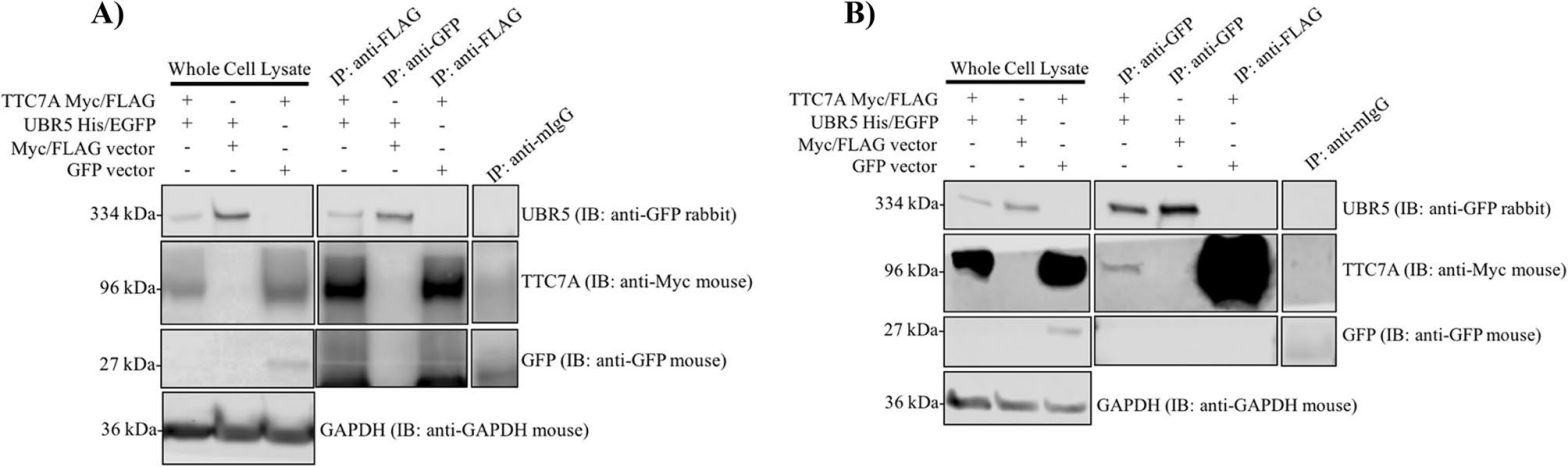
UBR5 and TTC7A interact with each another in HEK 293 T cells. (**A**) UBR5 co-IPs with TTC7A. n = 3. (**B**) TTC7A co-IPs with UBR5. Full length blots for (**A**) and (**B**) are presented in Supplementary Figures 3 and 4 respectively. n = 3.

**Figure 5:**
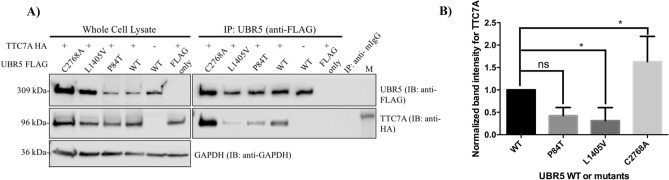
UBR5 L1405V mutant shows significantly reduced binding to TTC7A in HEK 293 T cells. (**A**) TTC7A co-IPs with differential binding affinities to UBR5 mutants. UBR5 plasmids were tagged with FLAG and the His tag removed. Lysate and IP samples were derived from the same experiment but ran on different blots. (**B**) UBR5 L1405V and C2768A show reduced and increased binding to TTC7A, respectively. Densitometry of western blot experiments from (**A**) were quantified and the values from all samples were made relative to the samples transfected with TTC7A and UBR5. Error bars indicate SD. *Denotes *p* < 0.05, ns = non-significant (student’s t-test). n = 4. Full length blots for (**A**) are presented in Supplementary Figure 5.

**Figure 6:**
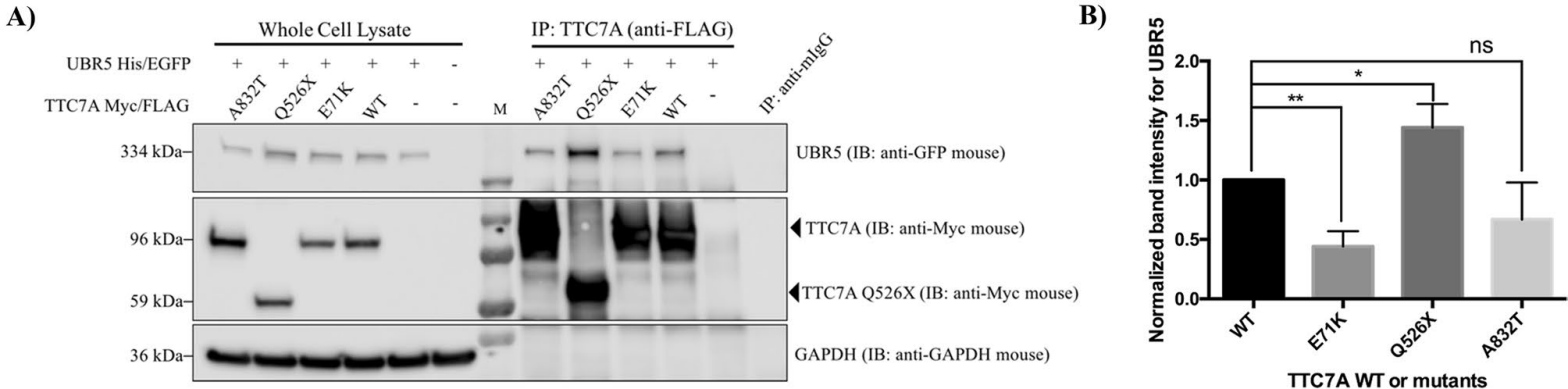
UBR5′s differential binding affinities to TTC7A VEO-IBD mutants. (**A**) UBR5 co-IPs with differential binding affinities to TTC7A VEO-IBD mutants. HEK 293 T cells were transfected with His/EGFP tagged UBR5 and Myc/FLAG tagged TTC7A WT, the mutants (A832T, Q526X, or E71K) or the tagged backbone vector only. (**B**) Densitometry of western blot from (**A**) was quantified and the values from all samples were made relative to the samples transfected with TTC7A and UBR5. Error bars indicate SD. **Denotes *p* < 0.01. n = 4. Full length blots for (**A**) are presented in Supplementary Figure 6.

Previously, tandem mass spectrometry (MS) using TTC7A WT and the E71K variants as bait identified UBR5 as a potential interactor of TTC7A^1^. To further validate these finding, we used co-immunoprecipitation (co-IP) studies in HEK 293 T cells and showed that UBR5 did co-IP with TTC7A (Fig. 4A) and TTC7A with UBR5 (Fig. 4B). TTC7A showed reduced co-IP with the identified UBR5 L1405V mutant protein, as compared with UBR5 WT (Fig. 5A,B). However, TTC7A showed increased co-IP with a catalytically dead UBR5 E3 ligase C2768A mutant in the HECT domain. Further analysis of the previously identified TTC7A VEOIBD mutants (E71K, Q526X, and A832T)^1^ also showed that UBR5 had reduced co-IP to the missense variants but increased co-IP with the Q526X truncation (Fig. 6A,B). These results validate the previously identified interaction between TTC7A and UBR5 and indicate a role of UBR5 in TTC7A signaling^1^. Full uncropped blots are available in Supplementary Figures 2–5.

## Discussion

We have recently demonstrated that 3% of pediatric IBD patients have monogenic forms of IBD^12^. Like the case presented here, many of these patients present at a very early age with severe disease that is difficult to treat^12^. The young age of our patient, the severity of disease requiring biologic therapy, and the presence of apoptosis on biopsy made this patient a strong candidate for genetic analysis to determine a potential monogenic cause of disease. We first screened for variants in genes known to be associated with VEOIBD, and as this patient was male we focused on X-linked genes associated with intestinal epithelial apoptosis including FOXP3 and XIAP, and also autosomal recessive genes including LRBA, ARPC1B, and TTC7A (see ^12,45^ for a complete list of genes associated with apoptosis). However, neither variants in these genes nor other genes associated with VEOIBD were identified. Therefore, we examined novel candidate genes and prioritized variant that were rare and damaging based on known biological function, animal models, and known interaction with previously identified VEOIBD genes.

*UBR5* was selected as a potential gene candidate based on evidence from our previous study utilizing tandem mass spectrometry to identify potential binding partners for TTC7A^1,18^. Bi-allelic deleterious variants in *TTC7A* were identified as a causal gene for severe intestinal and immune disease with high penetrance^1,13–16,18,21–24,46–49^. Many TTC7A-deficient patients present with clinical features associated with monogenic IBD or VEOIBD^1,14,15,18,21,22,47^. A key pathological feature of TTC7A deficiency is increased intestinal epithelial cell apoptosis^1,13,18,47^ that is not commonly found in typical non-genetic forms of IBD. Although, our patient did not have the severe immunodeficiency and intestinal stricturing disease observed in severe loss-of-function TTC7A mutations, he did have apoptotic colonic disease associated with the less severe form of the disease caused by hypomorphic TTC7A mutations.

With any potential novel VEOIBD variants, functional studies are required to demonstrate a potentially causative defect. Here we used co-IP experiments to validate our genetic studies and showed that variants in both TTC7A and UBR5 reduce co-IP. Previous studies of *Ubr5*^*-/-*^ mice demonstrated widespread apoptosis by E9.5 embryonic development stage^50^. UBR5 is a HECT E3 ubiquitin ligase that is found to be mutated or amplified in various cancer types (including colorectal cancer^51^), to inhibit intestinal apoptosis^35^ and UBR5 knockdown resulted in increased apoptosis in ovarian cells^52^. Pathological examination of biopsies from patients with both TTC7A and UBR5 variants demonstrate that UBR5 has low expression in the healthy gut but is highly upregulated in TTC7A-deficiency patient indicating a possible compensatory mechanism. Also, in our VEOIBD patient with *UBR5* variants, there was increased apoptosis in both epithelial and immune cells. As TTC7A is expressed in both epithelial and immune cells, this may be due to dysregulation of TTC7A-PI4K signalling but as UBR5 has a role in DUBA signalling in T-cells, this may contribute to disease progression^53^. It is interesting to speculate that our patient may benefit from treatment with Leflunomide as it was recently shown in a preclinical study as a potential therapy for TTC7A-deficiency^54^.

Our studies suggest that UBR5 may be associated with VEOIBD; however, there are a number of important limitations. First, despite searching a number of VEOIBD databases, we were unable to identify a second patient with bi-allelic variants in UBR5. UBR5 does not have any homozygous loss-of-function variants on gnomad (https://gnomad.broadinstitute.org/) suggesting that loss of function is detrimental and often it takes time to identify additional patients. Second, our functional studies using tandem mass-spec and co-immunoprecipitation experiments have shown that TTC7A and UBR5 appear to interact; however, we did not determine the precise role of UBR5 in TTC7A signaling. Therefore, further study into the function of this putative interaction and the relevant cell type(s) will be critical in our understanding of the disease pathogenesis.

## Methods

### Next-generation sequencing and data analysis

WES was performed in collaboration with the Regeneron Genetics Center (RGC) on this proband and his unaffected parents who were enrolled and consented in our NEOPICS partnership (https://www.neopics.org/). Exome capture was carried out using the NimbleGen VCRome 2.1 and sequencing was done using an Illumina HiSeq 2500 platform with paired-end 75 bp reads. Sequencing reads were aligned to human reference genome (GRCh38). Variants were called using the Genome Analysis Toolkit (GATK) (pmid:20644199) and the generated VCF files were subsequently annotated with snpEff (pmid:22728672). Polymorphisms reported in public databases with Minor Allele Frequency (MAF) > 1% and synonymous variants were filtered out. Potential pathogenicity protein-coding variants were prioritized using evolutionary conservation and various prediction tools (SIFT, PolyPhen2, Mutation Taster) from dbNSFP^55^. Inheritance modeling was carried out using GEMINI software^56^ (https://gemini.readthedocs.io/en/latest/) to identify variants that fit autosomal recessive, de novo, and X-linked inheritance patterns.

### Patient data availability

The identified UBR5 variants of our patient will be submitted to the ClinVar^57^ database (https://www.ncbi.nlm.nih.gov/clinvar/) upon publication. Information on the raw whole-exome sequencing data will not be published to protect research participant privacy.

### Sanger sequencing

Sanger sequencing was performed in the patient and parents to validate the compound heterozygous variants identified by WES. The genetic details for the variants are listed below:

NM_015902 (Homo sapiens ubiquitin protein ligase E3 component n-recognin 5 (UBR5), transcript variant 1, mRNA).

P84T: c.250c>a; dbSNP rs143719892; GRCh37 8:103372833 G>T; exon4.

L1405V: c.4213c>g; not in dbSNP; GRCh37 8:103306319 G>C; exon33.

The following primers were used to sequence P84T: forward TGGTAGAGTTTGCAGGATTGG (sense), and reverse TGATAACTGACTCCTCTGCTACT (anti-sense). The following primers were used to sequence L1405V: forward CCAAGGACTGTGGGACAAA (sense), and reverse CTCTTGCCACTGAACGTAGAA (anti-sense).

### Plasmid constructs

pCMV-Tag2B EDD and C2768A were a gift from Darren Saunders & Charles Watts (Addgene plasmid # 37188 and 37189 respectively)^43^. These plasmids were modified by deleting the His tag on the C-terminus of the cDNA for the experiment in Fig. 5. UBR5 mutant plasmids were created from the pCMV-Tag2B EDD WT plasmid by ACGT Corporation (Toronto). pEGFP-C1 EDD was also a gift from Darren Saunders & Charles Watts (Addgene plasmid # 37190)^43^. TTC7A WT and mutant plasmids (E71K, Q526X and A832T) with *myc-DDK* tags were previously generated^1^. Another TTC7A plasmid was constructed with pLJM1-EGFP entry vector with the deletion of the EGFP from the vector. A N-terminus *HA-*tagged TTC7A cDNA was subcloned into pLJM1 entry vector. pLJM1-EGFP backbone vector (or GFP vector) was used as a control for HA tagged TTC7A plasmids.

### Co-immunoprecipitation assay

HEK 293 T cells were grown on 10 cm plates and transfected with various combinations of plasmids using PolyJet (SignaGen Laboratories) according to standard protocols. 48 h post-transfection, cells were lysed with lysis buffer (150 mM NaCl, 50 mM HEPES, 1% Triton-X, 10% glycerol, 1.5 mM MgCl_2_ and 1.0 mM EGTA) supplemented with protease inhibitors (1 mM PMSF, 1 mM P2714, 2 mM Na_3_VO_4_ and 5 mM NaF). Lysates were not precleared before beginning the immunoprecipitation except for Fig. 5A experiment. 1 mg of lysate was immunoprecipitated with anti-FLAG beads (Sigma Aldrich or BioLegend) or anti-GFP beads (BioLegend) for 2 h at 4 °C. Negative control included lysates of all samples pooled with protein G beads (BioLegend) and 2 μg of mouse IgG antibody. Beads were washed 3 times with the same lysis buffer with protease inhibitors used to lyse the cells. Bound proteins were eluted using 35 μL of 2X SDS protein sample buffer (40% glycerol, 240 mM Tris/HCl, 8% SDS, 0.4% bromophenol blue, 5% beta-mercaptoethanol). 50 μg of lysate with 1-2X sample buffer and 30 μL of IP sample (15 μL for experiments in Fig. 4A,B) was loaded into 8–10% SDS-PAGE and subject to western blot analysis. Semi-dry transfer was performed using a Bio-Rad machine and nitrocellulose membrane. All experiments were performed in triplicate unless otherwise stated.

### Statistical analysis

Co-IP: Odyssey FC (LI-COR Biosciences), a chemiluminescence scanner, was used for imaging of the western blot. Densitometry of the western blot was obtained by ImageStudioLite (LI-COR Biosciences) and quantified by making the ratio of the IP band for a protein in a sample relative to the lysate band in the same sample. For each sample, the IP/lysate ratio obtained for TTC7A for each sample was divided with IP/lysate ratio for UBR5 from the same sample. For each sample, the values obtained for TTC7A/UBR5 ratios of IP/lysate were made relative to TTC7A WT + UBR5 transfected sample. Vice versa was done for Fig. 5A to generate Fig. 5B. GraphPad Prism 5.0 software (GraphPad Software, San Diego, CA) was used to create the graph and student’s *t*-test was performed for statistical analysis of the variables of interest. All experiments were n = 3 unless otherwise stated.

### IF histochemical staining on formalin-fixed paraffin-embedded (FFPE) sections

Colon-sigmoid mucosa tissue samples were retrieved from the Division of Pathology, The Hospital for Sick Children. These samples include healthy control, IBD control with normal GI histology and without validated variants from infants. Patient’s biopsies from Spain (UBR5 patient) and BC (TTC7A patient^44^) infants were obtained with Ethics approval and informed consent as previously summarized^58^.

#### Dual immunofluorescent histochemical staining

The details for IF staining on FFPE section procedure are published^58^. Briefly, as a first step, paraffin was removed using Xylene, and afterwards rehydrated with different percentages of ethanol. Antigen retrieval was performed with high-pressure cooking in EDTA–borax buffer (1 mM EDTA, 10 mM borax (sodium tetraborate, Sigma, St Louis, MI, USA), 10 mM boric acid (Sigma) with 0.001% Proclin 300 (Supleco, Bellefonte, PA, USA) at pH 8.5. To block non-specific staining, the slides were incubated for 1 h at room temperature in 4% BSA in 1X phosphate-buffered saline (PBS, Multi Cell) 20% normal donkey serum. A properly diluted primary antibody, for example, rabbit anti-TTC7A (see Table 1 for detail) polyclonal antibody and anti-cyto-structure mouse monoclonal antibody (Abcam Inc. Toronto, Ontario, see Table 1) incubation was performed overnight at 4 °C. On the following day, stained slides were washed three times for 5 min with 1X PBS. Secondary antibody, donkey anti-rabbit IgG Fab2 fragment-Rhodamine conjugate mixed with donkey anti-mouse IgG Fab2 fragment-FITC conjugate (Jackson Immuno Research Lab, West Grove, PA) incubation was performed at room temperature in darkness for 2 h, and slides were washed afterwards three times for 10 min in darkness. As a nuclear counterstain reagent, RedDot2 far red fluorescence (Biotium Inc. Fremont CA) was used at a dilution of 1:200. Finally, sections were mounted overnight with Vector shield fluorescence mounting medium (Vector Labs, Burlington, ON).

**Table 1:** Antibodies.

Primary antibody	Blocking	1° antibody (dilution)	Source of 1° antibody	Catalogue number	2° antibody (dilution)
**Western blot**					
Anti-GFP mouse	5% skim milk	1:500–1:1000	Invitrogen	–	1:3000
Anti-GFP mouse	5% skim milk	1:500–1:1000	Biolegend	902602	1:3000
Anti-Myc mouse	5% skim milk	1:1000	Millipore	05-724	1:3000
Anti-Myc rabbit	5% skim milk	1:1000	Cell signaling technologies	06-549	1:3000
Anti-GAPDH mouse	5% skim milk	1:1000–1:3000	Abgent	AM1020b	1:3000
Anti-HA mouse	5% skim milk	1:1000	Biolegend	MMS-101P	1:3000
Anti-HA rabbit	5% skim milk	1:1000	Cell signaling technologies	3724S	1:3000
Anti-FLAG mouse	5% skim milk	1:1000	Origene	AM26389PU-N	1:3000
Anti-FLAG rabbit	5% skim milk	1:1000	Cell signaling technologies	2368S	1:3000
**Immunohistochemistry (IHC) staining**					
Anti-UBR5 mouse mAb	20%NDS in 4%BSA-PBS	1:100	Millipore Sigma	MABF1110	1:100
Anti-TTC7A rabbit pAb	20%NDS in 4%BSA-PBS	1:200	NOVUS	NBP1-93601	1:200
Anti-Caspase 3 rabbit pAb	20%NDS in 4%BSA-PBS	1:200	NOVUS	NB100-56113	1:200
Anti- β-catenin rabbit pAb	20%NDS in 4%BSA-PBS	1:200	BP transduction	610154	1:200

#### Confocal microscopy

Double/triple-immunostained sections were imaged using a Leica confocal laser scanning microscope (model TCS-SP8) and LAS-AF software (Leica Microsystems, Wetzlar, Germany), as previously reported^59^. The variable excitation wavelengths of the krypton/argon laser were 488 nm for fluorescein isothiocyanate conjugate, 568 nm for Texas Red complex, and 695 nm for Alexa Fluor 680 conjugate/RedDot 2 (nuclear counterstaining). Image processing, including color resolution, color separation, and merging of fields, were carried out using Adobe PhotoShop CS5 software (Adobe Systems Incorporated, San Jose, CA, USA).

#### Morphometric and colocalization analysis

The NIH ImageJ software was used with appropriate algorithms to analyze the degree of co-occurrence and correlation for TTC7A and UBR5 in the images. A total of 100 cells were selected that showed positive staining for both, TTC7A and UBR5, from 5 different areas within each slide. The JACoP plugin^60^ for ImageJ was applied in these cells for obtaining Pearson and Mander’s coefficients. Previously reported algorithms^61^ were used to determine co-localization and the associated statistics are reported in Supplementary Table 3. Linear regression/correlation and the *t*-test were used for the statistical/correction analysis to report on colocalization.

### Helsinki guidelines

All human experiments followed the Helsinki Guidelines.

### Informed consent

Informed consent was obtained from the participant’s parents and the study had local ethics board approval at Hospital Regional Universitario de Málaga, Málaga, Spain and Hospital for Sick Children, Toronto, Canada (Research Ethics Board: REB1000024905).

## Supplementary information

Supplementary Information 1.
