# Supplementary material for: The E3 ubiquitin ligase UBR5 interacts with TTC7A and may be associated with very early onset inflammatory bowel disease
Source: Sci Rep. 2020 Oct 29;10:18648. doi: 10.1038/s41598-020-73482-6 (PMC7596066; doi:10.1038/s41598-020-73482-6)
Supplement: Supplementary file 1 — Supplementary Information 1. [file 41598_2020_73482_MOESM1_ESM.docx]

**The E3 ubiquitin ligase UBR5 interacts with TTC7A and may be associated with Very Early Onset Inflammatory Bowel Disease**

Neel Dhingani^1,2^, Conghui Guo^2^, Jie Pan^2^, Qi Li^2^, Neil Warner^2^, Sasha Jardine^1,2^, Gabriella Leung^2^, Daniel Kotlarz^3^, Claudia Gonzaga-Jauregui^4^, Christoph Klein^3^, Scott B Snapper^5^, Víctor Manuel Navas-López^6^*****, Aleixo M Muise^1,2,7,8^*****

**Supplementary Figures:**


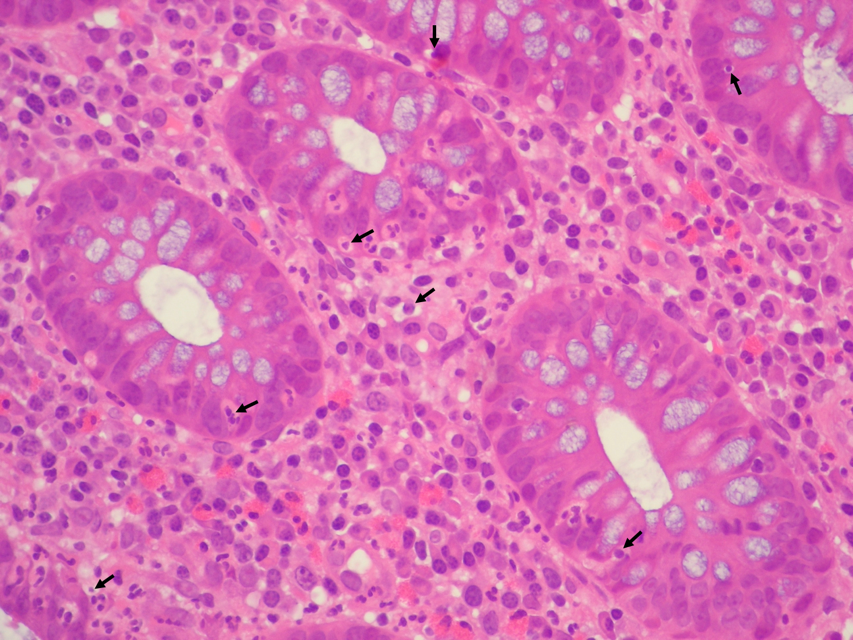

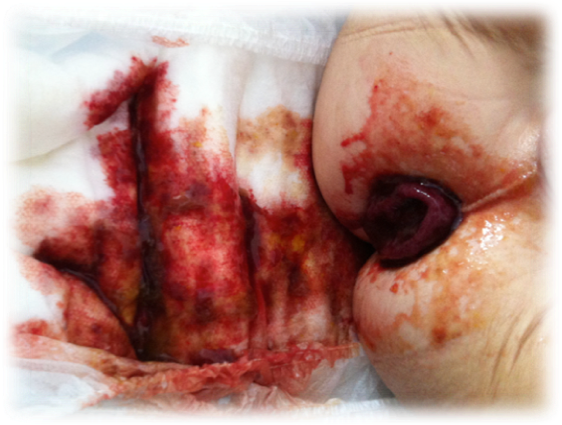


**A)**


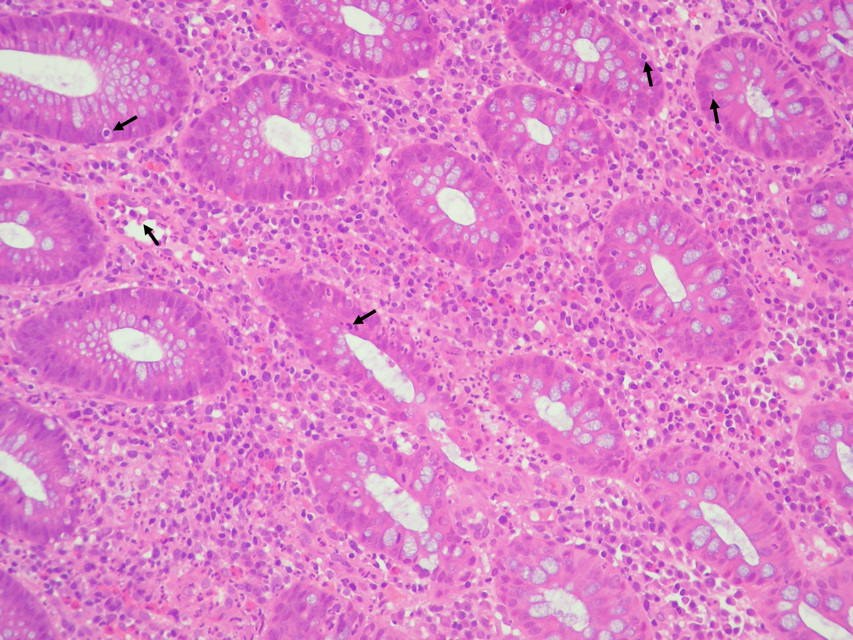


20X

40X

**B)**


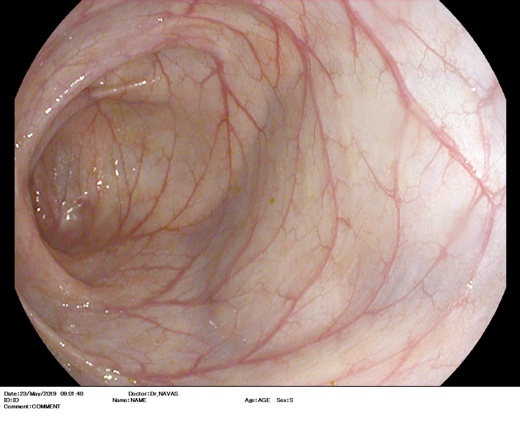


**C)**


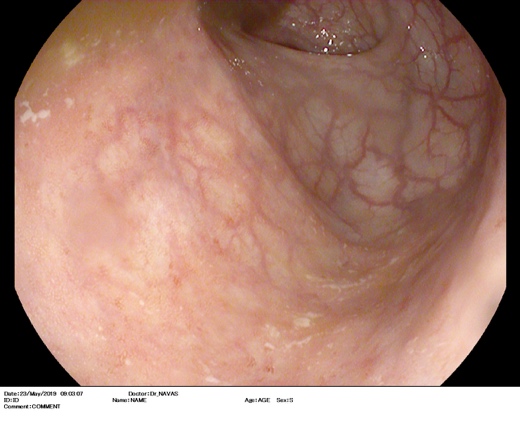


**D)**

**Supplementary Figure 1:** Clinical features of the UBR5 patient. A) Rectal relapse seen in UBR5 patient at the onset of VEOIBD diagnosis (at patient’s age of 2 years, 9 months). B) H&E staining of inflamed rectum section from the UBR5 patient. Apoptotic cells are indicated by an arrow. Endoscopy of the UBR5 patient at age of approximately 9 years shows C) mild proctitis, but overall, normal mucosa of sigmoid colon and D) shows transition area between rectum and sigmoid colon.

**
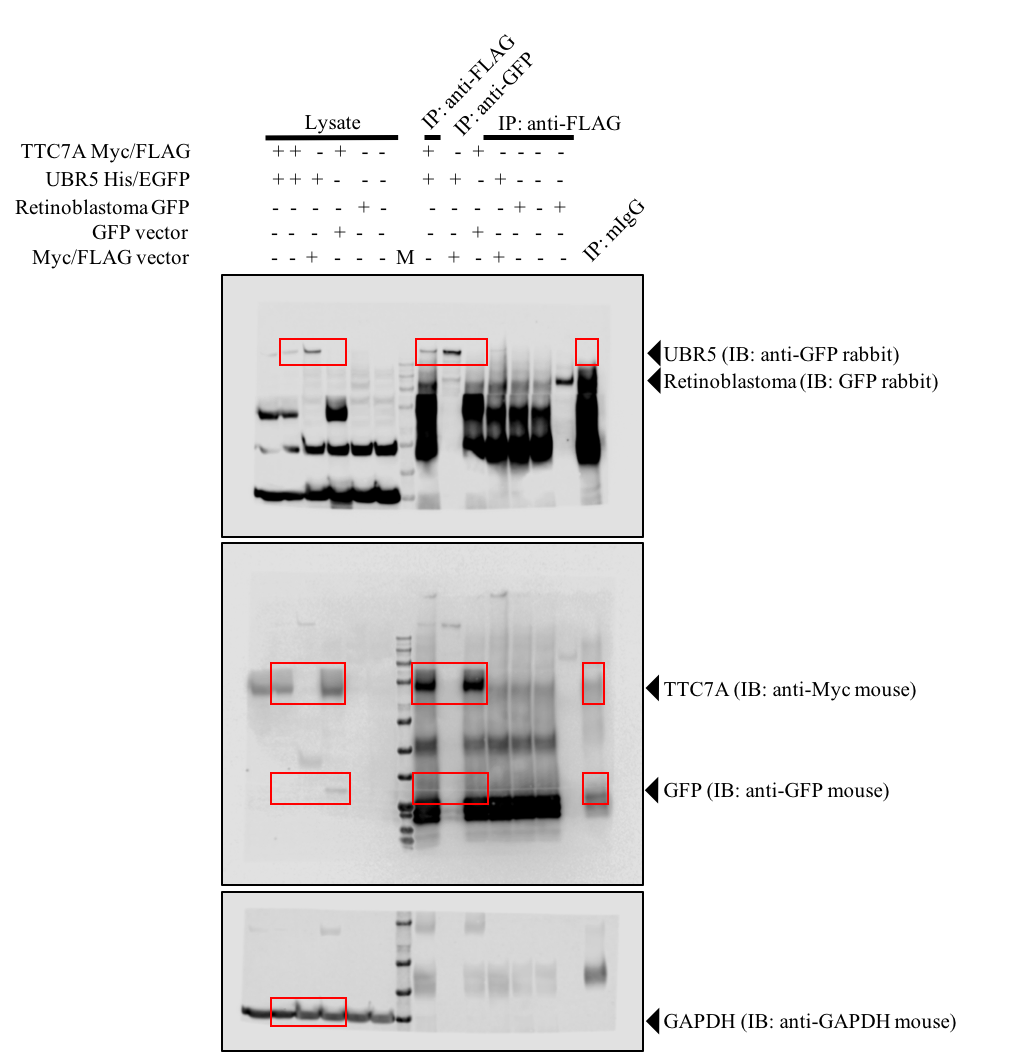
**

**Supplementary Figure 2:** Full length blots for Figure 4A.


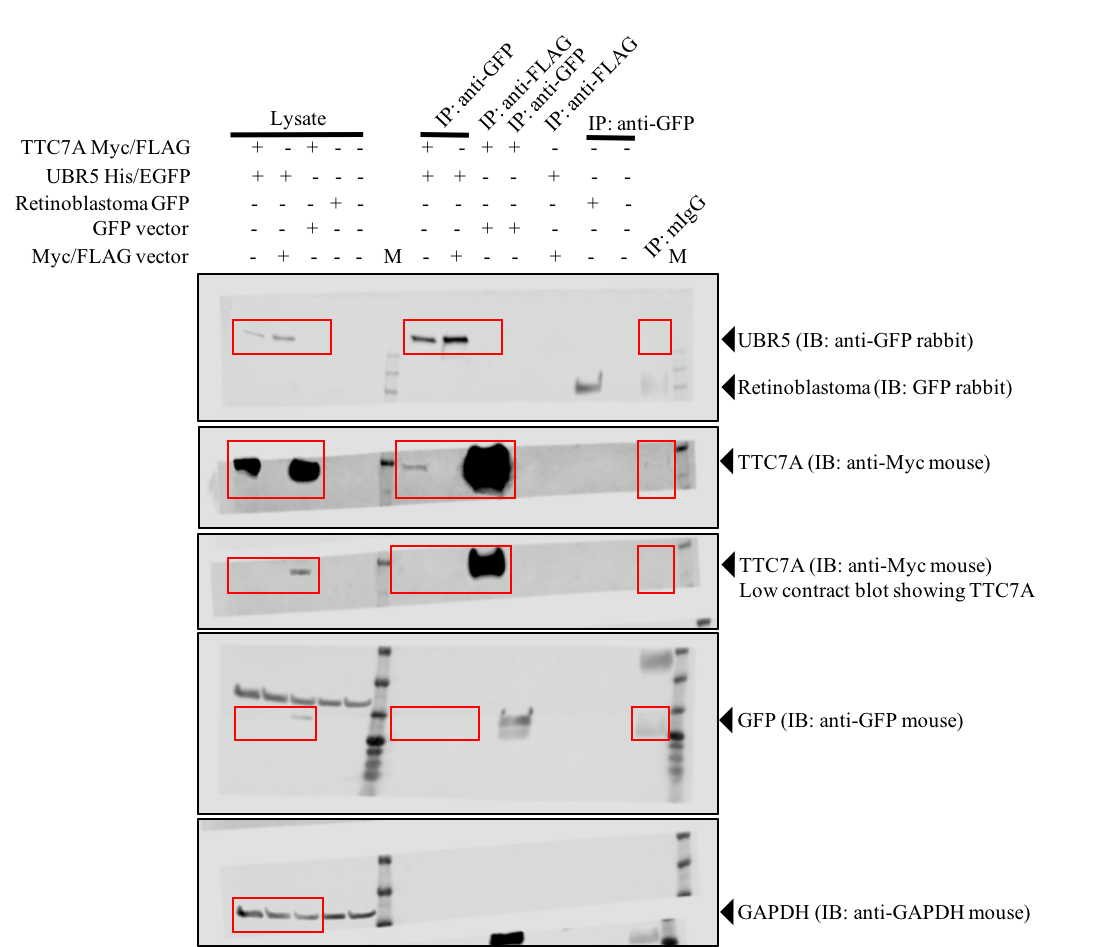


**Supplementary Figure 3:** Full length blots for Figure 4B.


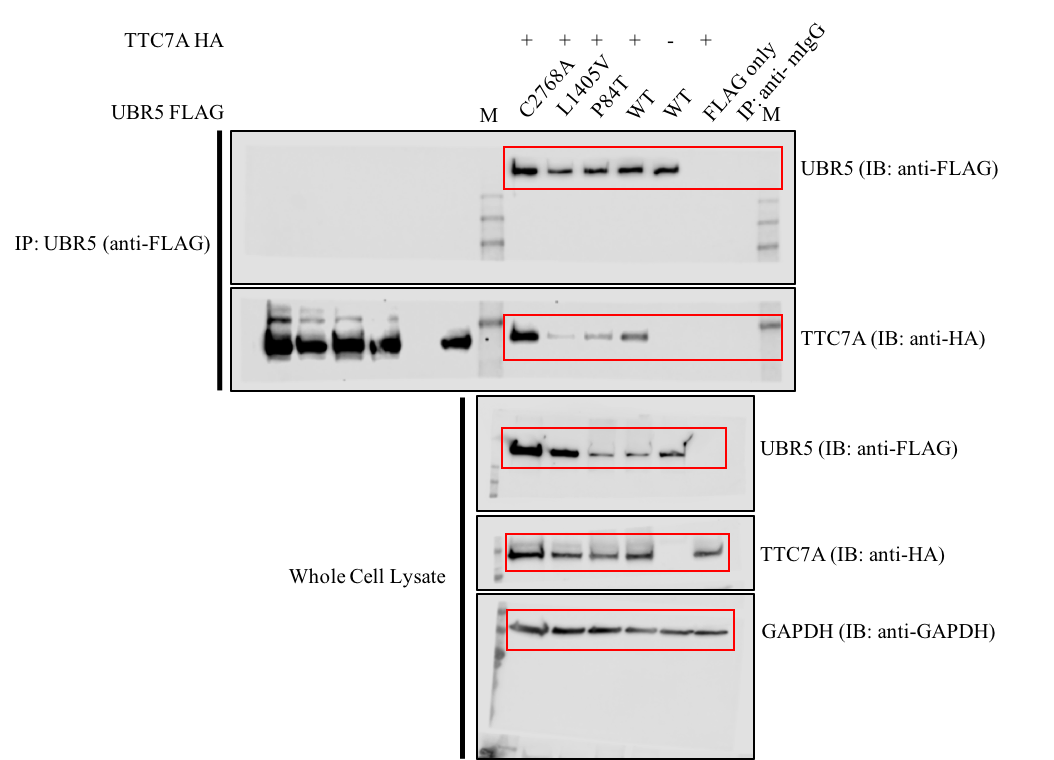


**Supplementary Figure 4:** Full length blots for Figure 5A.


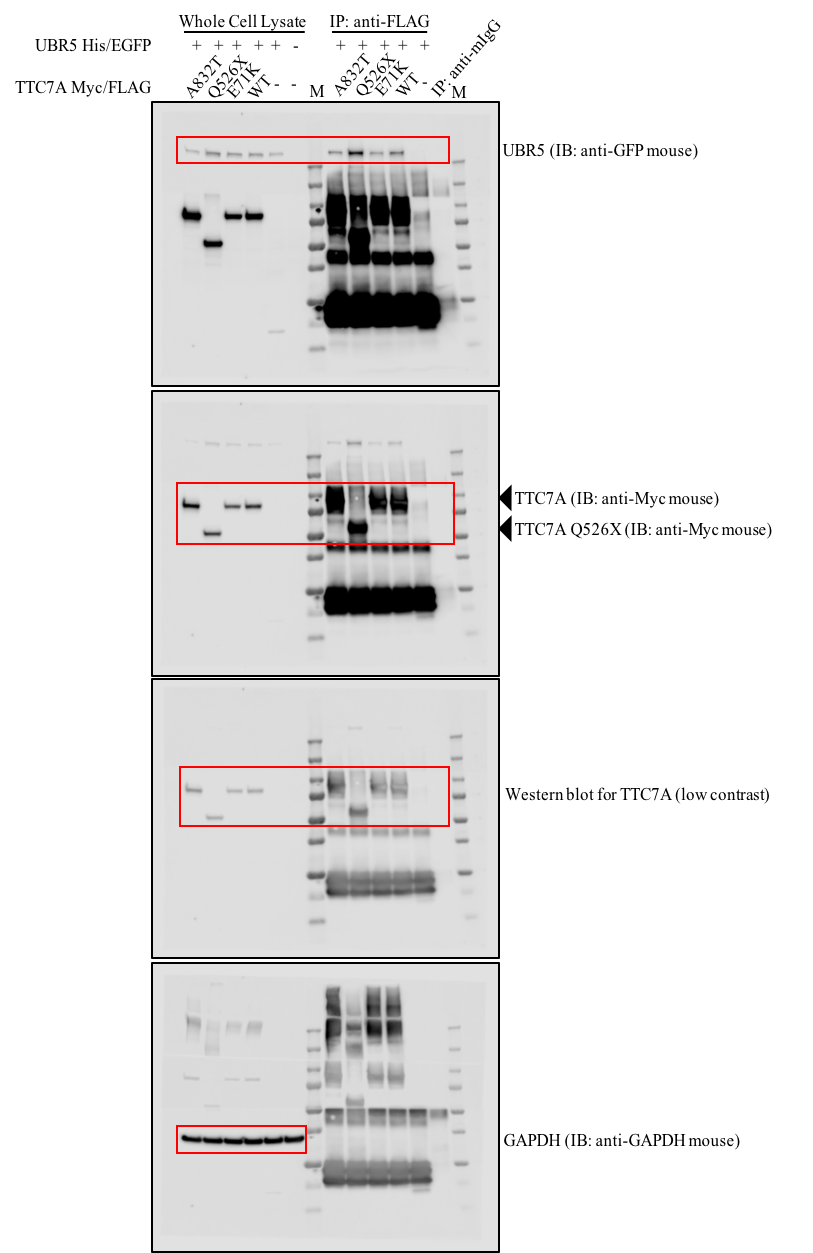


**Supplementary Figure 5:** Full length blots for Figure 6A.

**Supplementary Tables:**

**Supplementary Table 1:** Blood work analysis of the UBR5 patient at both, time of diagnosis and endoscopy. Red font indicates values which are above or below the reference values.

| Laboratory results | Reference | Baseline  (April 2013) | Time of scope  (January 2015) |
| --- | --- | --- | --- |
| Faecal calprotectin, μg/g | < 50 | 950 | N/A |
| C-reactive protein  (CRP), mg/L | < 5 | 8.5 (upper limit of normal [ULN] = 5) | <5 |
| Erythrocyte sedimentation rate  (ESR), mm/h | < 30 | 32 | 7 |
| Albumin, g/dL | 3.80 - 5.40 | 2.5 | 4.8 |
| Vitamin D, ng/mL | 30.0 - 60.0 | 17.3 | 27.2 |
| Hemoglobin, g/dL | 11-14.5 | 9.7 | 12.5 |
| Hematocrit  (Htc), % | 30-46 | 30.1 | 38 |
| White blood cells  (WBC), x10^9^/L | 4-11.5 | 8.9 | 8.9 |
| Neutrophils, x10^9^/L | 1.8-8.0 | 4.3 | 4.7 |
| Lymphocytes, x10^9^/L | 0-6-4.6 | 3.1 | 3.2 |
| Platelets, x10^9^/L | 140-450 | 517 | 414 |
| Aspartate aminotransferase  (AST)/Alanine aminotransferase (ALT), U/L | AST 13-40  ALT 7-40 | 52/31 | 28/17 |
| p- antineutrophil cytoplasmic antibodies (ANCA) | -ve | +ve | N/A |
| Anti-Saccharomyces cerevisiae antibodies (ASCA) | -ve | -ve | N/A |
| Immunoglobulin A (IgA), mg/dl | 70-400 | 99 | 171 |
| Immunoglobulin G (IgG), mg/dl | 700-1600 | 1550 | 1030 |
| Immunoglobulin M (IgM), mg/dl | 40-230 | 116 | 101 |

**Supplementary Table 2:** List of all the prioritized variants from WES trio analysis for UBR5 patient.

| **Chromosome** | **Gene** | **Codon Change** | **Amino Acid (AA) Change** | **AA Length** | **Type of Mutation** | **Impact** | **Polyphen Prediction** | **Sift Prediction** | **Combined Annotation Dependent Depletion (CADD)** | **Exome Aggregation Consortium (ExAC)** | **Gnomad** | **Inheritance Model** |
| --- | --- | --- | --- | --- | --- | --- | --- | --- | --- | --- | --- | --- |
| chr10 | FAM21A | Ccc/Acc | P/T | 1177/1320 | missense | MED | Probably damaging | tolerated | 22.29999924 | None | None | AR |
| chr9 | SPATA31A6 | cCc/cTc | P/L | 420/1343 | missense | MED | Probably damaging | deleterious | 24 | None | None | AR |
| chr22 | MYO18B | Cgt/Tgt | R/C | 1748/2568 | missense | MED | Possibly damaging | deleterious | **35** | 0.0011 | 0.0011 | CH |
| chr22 | MYO18B | Cgg/Tgg | R/W | 2450/2568 | missense | MED | Probably damaging | deleterious | **33** | 0.0049 | 0.0044 | CH |
| chr8 | UBR5 | Cta/Gta | L/V | 1405/2799 | missense | MED | Probably damaging | deleterious | 24.60000038 | None | None | CH |
| chr8 | UBR5 | Cct/Act | P/T | 84/2799 | missense | MED | Probably damaging | tolerated | 23.5 | 9.89E-05 | 3.23E-05 | CH |
| chrX | THOC2 | cGc/cAc | R/H | 1409/1593 | missense | MED | Probably damaging | deleterious | 32 | None | None | X-linked |
| chr17 | OMG | aaT/aaA | N/K | 143/440 | missense | MED | Probably damaging | deleterious | 23.60000038 | None | None | DN |
| chr17 | SSH2 | tCa/tGa | S/* | 670/1450 | Stop gained | HIGH | N/A | N/A | 40 | None | None | DN |

**Supplementary Table 3:** Data from statistical analysis of colocalization between TTC7A and UBR5 from Figure 3. 100 cells were selected for colocalization analysis that demonstrated positive immunofluorescence signal for both, TTC7A and UBR5, from 5 different areas. Pearson coefficient are shown through r value^1^ which measures linear correlation (yellow color) between UBR5 (shown in red color) and TTC7A (shown in green color). P value shows statistical significance of r values from each biopsy section. NS = non-significant. Mander’s colocalization coefficient, M1, represents TTC7A’s colocalization with UBR5 and M2 shows vice versa.

| Biopsy sections | Pearson coefficient | P value | M1 | M2 |
| --- | --- | --- | --- | --- |
| Healthy control | r = 0.958 | <0.01 | 0.554 | 0.548 |
| IBD w/o mutation | r = 0.921 | <0.01 | 0.614 | 0.678 |
| TTC7A variant | r = 0.001 | NS | 0.114 | 0.071 |
| UBR5 variant | r = 0.833 | <0.01 | 0.512 | 0.567 |

**References**

1 Villalta, J. I. *et al.* New Algorithm to Determine True Colocalization in Combination with Image Restoration and Time-Lapse Confocal Microscopy to Map Kinases in Mitochondria. *PLOS ONE* **6**, e19031, doi:10.1371/journal.pone.0019031 (2011).
